# Nurses’ Experiences of Rebel Nurse Leadership in Psychiatric Care: A Qualitative Interview Study of Positive Deviance

**DOI:** 10.1155/jonm/2777470

**Published:** 2026-06-28

**Authors:** Sebastian Gabrielsson, Git-Marie Ejneborn Looi, Annika Ahlblom, Ylva Kortekaas Genstrand, Anneli Björkdahl, Jessica Östling, Susanne Berg, Nina Lakso, Mattias Larsson, Johanna Salberg

**Affiliations:** ^1^ Department of Health, Education, and Technology, Luleå University of Technology, Luleå, Sweden, ltu.se; ^2^ Department of Health Sciences, The Swedish Red Cross University, Huddinge, Sweden

**Keywords:** content analysis, mental health nursing, positive deviance, psychiatric care, qualitative study, rebel nurse leadership

## Abstract

Nurses play a key role in person‐centered psychiatric care by supporting patient recovery, fostering independence, and building trusting relationships. However, organizational rules may conflict with nurses’ ethical values, leading to moral distress, burnout, and resignation. Drawing on the theory of positive deviance, previous research has identified how nurses may engage in positive rebel leadership, leading and practicing nursing in ways that diverge from prevailing norms, rules, codes of conduct, and workplace strategies. This study explores how such leadership in psychiatric care can support professional standards and improve patient outcomes. The aim was to describe nurses’ experiences of rebel nurse leadership in psychiatric care. A qualitative descriptive design was employed, using semistructured individual interviews with 33 nurses experienced in psychiatric care. A qualitative content analysis and meta‐synthesis were conducted, with the support of generative artificial intelligence in the synthesis process. The results describe nurses’ experiences of rebel nurse leadership in psychiatric care as *taking responsibility, guided by professional competence and an internal ethical compass; leading change and challenging hierarchies to enable holistic care that respects patients’ rights and dignity; relying on the support of colleagues and management, while facing the risk of exclusion.* The study shows how rebel nurse leadership, grounded in professional competence and ethical conviction, might support person‐centered and high‐quality psychiatric care. The findings illustrate how such leadership emerges in response to organizational norms that constrain nursing practice and frame acts of resistance as expressions of professional responsibility. The study emphasizes the importance of supporting nurses’ autonomy to enable improvements in care quality and patient outcomes.

## 1. Introduction

While mental health nursing endures in name, nurses in psychiatric care are often viewed as subordinate to the medical profession, with their work reduced to tasks and procedures [1, 2]. In such environments, employers may prefer compliant “zombies” over reflective, confident professionals who are more likely to challenge the status quo. By taking responsibility for psychiatric care, nurses can make a meaningful contribution to person‐centered practice [3]. Drawing on the concepts of positive deviance and rebel nurse leadership, this study explores how nurses, by adopting a critical stance and going against the grain, can strengthen and develop mental health nursing within the context of psychiatric care.

## 2. Background

Nurses play an important role in supporting patients on their unique journey toward recovery [4, 5]. Recovery‐oriented nursing enables patients to gradually take responsibility for their own lives [4] and to live a meaningful and satisfying life, even with the limitations that illness may impose [5]. A trusting relationship forms the foundation of quality nursing care and is essential for nurses to develop an understanding of each individual [6]. Such relationships can be fostered when nurses take professional responsibility for both nursing care and seeing the patient as a person [3].

Nurses’ own professional standards clearly articulate an expectation that nurses should “assume the major leadership role in determining and implementing evidence‐informed, acceptable standards of clinical nursing practice, management, research and education” ([7], p. 15). However, psychiatric and mental health nurses are frequently regarded as assistants rather than as members of an autonomous profession, which may be attributed to the absence of a clear description of the nurse’s role in psychiatric care [1, 8]. This lack of clarity may prevent nurses from working to their full potential [8]. Nurses in psychiatric settings often encounter strict organizational rules and codes of conduct that limit their ability to practice independently and fully exercise their professional expertise [9]. This constrained view of the nursing role appears to be rooted in a medical paradigm, in which the nurse’s work more closely resembles that of a medical assistant [1, 10].

Nurses working in psychiatric care report feeling pressured to carry out actions that they do not believe are in the patient’s best interests [11, 12], often in contexts where the nursing perspective is overlooked in favor of a medical one [1, 10]. As a result, nurses may experience internal conflict when the care provided conflicts with their ethical values and the ideals of good psychiatric care can no longer be upheld [11, 13–15].

For some nurses, this ethical stress can lead to decisions to resign from their positions [12], to empathic exhaustion [16], or to negative consequences for their own health and well‐being [17]. For others, the internal conflict may trigger a form of resistance: actions taken in the patient’s best interest that nonetheless deviate from organizational guidelines. When nurses perceive that such deviant actions truly benefit the patient, they may feel a sense of pride and professional integrity in their decision [13].

### 2.1. Positive Deviance and Rebel Nurse Leadership

Positive deviance refers to behaviors or strategies that constructively deviate from established norms and lead to improved outcomes. In health care, positive deviance strategies are considered to have the potential to enhance the quality of care, as the solutions are likely to be sustainable, acceptable to staff, and feasible within existing resources [18, 19]. However, previous research indicates that deviating acts in nursing practice may often be quick fixes rather than drivers of sustainable change [20].

In the nursing literature, de Kok et al. [11, 15] conceptualize nurses’ positive deviance as *rebel nurse leadership*, describing it as a form of leadership in which nurses lead and practice nursing in ways that diverge from prevailing norms, rules, codes of conduct, and organizational strategies. These actions are undertaken in a positive and responsible manner, grounded in professional knowledge and aligned with organizational goals. Rebel nurses also inspire others to act differently in ways that improve outcomes for both patients and healthcare organizations. To achieve sustainable change, rebel nurses need supportive managers [20].

The phenomenon of rebel nurse leadership has not previously been explored in the context of psychiatric care. *Good rebels* are described as creative and exploratory, firmly rooted in convictions based on professional knowledge and experience [15]. They are willing to voice these convictions to persuade and motivate others. *Good rebel leadership* is described as contagious, inspiring others to reflect critically and to pursue positive change.

In contrast, *bad rebel leadership* is exemplified by nurses who complain, grumble, or resist participation in changes designed to benefit patients. In such cases, resistance is not rooted in professional judgment or patient interest. Good rebel leaders, by contrast, can justify their refusal to conform based on the best interests of the patient and can propose constructive alternatives [15].

### 2.2. Rationale

Rebel leadership challenges conventional guidelines and norms within the organization, with the intention of meeting patients’ needs, upholding professional standards, and providing high‐quality care [11]. These deviations are not in conflict with organizational goals; however, they can be difficult to detect, as they often occur under the radar, for example, due to concerns about potential reprimands [11, 15].

### 2.3. Aim

This study aimed to explore nurses’ experiences of rebel nurse leadership in psychiatric care.

## 3. Methods

### 3.1. Design

The study employed a descriptive design and qualitative approach, with data collected through semistructured individual interviews [21]. It is a meta‐synthesis of four distinct datasets, each originally gathered from participants in different clinical settings and initially analyzed separately. Students enrolled in specialist education in mental health nursing were invited to contribute to data collection and analysis as part of their master’s thesis.

### 3.2. Participants and Procedure

Both bachelor‐level registered nurses and registered nurses specialized in mental health nursing were eligible to participate in the study. Participants were recruited through the researchers’ professional networks and social media platforms, such as Facebook, Twitter, and Instagram. To be eligible for inclusion, participants were required to have experience in one or more of the following areas: general psychiatric inpatient care, child and adolescent psychiatric inpatient care, forensic psychiatric care, or general psychiatric outpatient care.

Nurses who were interested in participating completed a web form that included detailed information about the study and the option to provide written informed consent. Potential participants were also given the opportunity to contact the researchers with any questions. In addition to the written information provided in the web form, oral information was given before the interviews. To ensure informed consent, additional oral consent was obtained at the time of the interviews.

A total of 51 nurses expressed interest in participating in the study. Two were excluded because their primary experience was in other subspecialties (addiction and psychosis). Eighteen nurses could not be reached or declined to participate. In total, 33 nurses were included in the study. They were either currently employed in or had their main experience from one of the following settings: general psychiatric inpatient care (*n* = 8), child and adolescent psychiatric inpatient care (*n* = 8), forensic psychiatric care (*n* = 8), or general psychiatric outpatient care (*n* = 9). Of the 33 participants, 24 were specialist nurses, and three were in training to become specialist nurses. Thirty‐one were identified as women and two as men. Their experience in psychiatric care ranged from 6 months to 30 years, with an average of 10 years and 4 months.

### 3.3. Interviews

Data were collected through individual semistructured interviews [21] focusing on nurses’ experiences of rebel nurse leadership in psychiatric care. Interviews were conducted in October and November 2023. An interview guide was used, based on the work of de Kok et al. [15], who in turn developed their guide from a systematic review [11]. The interview guide was translated into Swedish for this study. We followed the structure of the original guide closely, with one exception: We chose to omit the “photo exercise” included by de Kok et al. [11], as it was not feasible within our data collection design.

The first three interviews were conducted by experienced researchers. This approach served as a way to pilot‐test the interview guide and ensure that the questions were coherent, relevant, and generated the type of data required for the study. The pilot phase did not lead to any changes. The remaining interviews were conducted by master’s degree students enrolled in specialist education in psychiatric nursing, under the supervision of experienced researchers. The interviews began with background questions, followed by an exploratory opening question (“What does rebel nurse leadership mean to you?”). Participants then received a brief introduction to the concept of rebel nurse leadership before responding to 10 questions about their own experiences, such as “Could you describe how rebel nurse leadership comes forward in your practice?” The interview concluded with closing questions. Throughout the interviews, follow‐up and clarifying questions were asked to deepen and clarify participants’ accounts.

The interviews lasted between 12 and 68 min, with an average duration of 39 min. Interviews were carried out primarily via digital platforms (Zoom or Teams), with one interview conducted by telephone and one in‐person, at the participants’ request. In all cases, the time and mode of interview were arranged in consultation with each participant. Both interviewers and participants ensured they were in a private and quiet location to minimize interruptions. All interviews were audio‐recorded and transcribed verbatim. Table 1 provides detailed information about the participants and interviews across the four settings.

**TABLE 1:** Detailed information about the participants and interviews across the four settings.

			General psychiatric inpatient care	Child and adolescent psychiatric inpatient care	Forensic psychiatric care	General psychiatric outpatient care
Participants	Number		8	8	8	9
	Age	Range (years)	36–65	28–61	27–43	33–57
		Average (years)	47	44	35	46
	Gender	Women	7	8	7	9
		Men	1	0	1	0
	Experience	As nurse (years)	4–30, avg. 14	6–30, avg. 16	0.5–19, avg. 9	5–21, avg. 12
		In psychiatric care (years)	2–30, avg. 11	6–27, avg. 12	0.5–19, avg. 9	1–20, avg. 11
	Qualification	Specialist nurse	5	7	3	9
		General nurse training to be specialist nurse	0	1	2	0
		General nurse	3	0	3	0

Interviews	Length	Range (minutes)	12–56	38–68	20–34	31–68
		Average (minutes)	37	49	28	41
	Medium	Digital	7	7	8	9
		Phone	0	1	0	0
		Physical	1	0	0	0
	Interviewers	Students	7	8	8	7
		Experienced researchers	1	0	0	2

### 3.4. Analysis

A qualitative content analysis [22–24] and meta‐synthesis were conducted. First, interview transcripts were organized according to participants’ primary subspecialty experience (general psychiatric inpatient care, child and adolescent psychiatric inpatient care, forensic inpatient care, and general psychiatric outpatient care) and analyzed separately. Second, the results of these individual analyses were combined and synthesized. The analysis took an inductive approach. Initially, all separate analyses focused on the manifest content and the development of categories and subcategories. In one of the initial analyses, the process was taken further by formulating themes and subthemes that captured latent content. The final synthesis focused on describing latent content through overarching themes.

The separate analyses followed the steps outlined by Graneheim and Lundman [23]. The transcripts were first read several times to establish a sense of the whole. Meaning units relevant to each study’s aim were then identified, condensed, and assigned descriptive codes. Throughout the analyses, the authors worked iteratively, moving back and forth between the raw text, condensed meaning units, codes, and developing categories, to ensure that important nuances were retained. Codes were grouped based on similarities and differences, forming categories that represented the manifest content of the data. In all initial analyses, the analytic process was carried out collaboratively, with categories refined through repeated discussions among the authors and with input from supervisors and other students. Together, these analyses yielded categories and themes that captured both commonalities and variation in participants’ experiences, while adhering to the methodological principles of qualitative content analysis. An example of the steps in the analytic process is provided in Table 2.

**TABLE 2:** Example of the analytic process from the separate analysis of interviews with nurses experienced in forensic psychiatric inpatient care.

Meaning unit	Condensed meaning unit	Descriptive code	Category
But I’m thinking that you experience, uh…that there is something you want to improve, and it doesn’t work within the framework of the norms that exist, or that you can do it better outside those norms.	You feel that there is something you want to improve that doesn’t work within the framework of the existing norms, or that you can do it better outside that framework.	Something you want to improve outside the existing norms and frameworks.	Daring to be an annoying colleague and question care cultures, norms, and challenge rules.

An initial synthesis was generated using GPT‐4 [25]. The following prompt was applied: “Present a metasynthesis of the attached qualitative research findings. The metasynthesis should describe nurses’ experiences of rebel nurse leadership in psychiatric care.” This provided a synthesis suggesting six themes with short descriptions. A second prompt was applied: “Use the attached findings to elaborate on each theme, providing examples and describing similarities and differences between experiences with nurse rebel leadership in the four different settings (outpatient psychiatric care, adult psychiatric inpatient care, child and adolescent psychiatric inpatient care, forensic psychiatric inpatient care).”

Using the concepts of abstraction and interpretation as described by Lindgren et al. [24], we understood the AI‐generated themes to represent a high level of abstraction but a low level of interpretation. The AI‐generated synthesis served as the basis for critical discussions and further iterative analysis of the combined results. This process involved repeated discussions within the research group, the formulation and revision of alternative themes, and their validation against the results from the separate analyses. Ultimately, the process resulted in three overarching themes with a lower level of abstraction and a higher degree of interpretation than the themes suggested by GPT‐4, describing nurses’ experiences of rebel nurse leadership in psychiatric care. Table 3 provides a comparison of the AI‐generated themes and final themes.

**TABLE 3:** Comparison of AI‐generated themes and final themes.

AI‐generated themes	Final themes
• Challenges and isolation in rebel leadership • Importance of support from peers and management • Impact on work environment and patient care • Personal attributes and professional responsibility • Emotional resilience and ethical commitment • Cultural and structural change	• Taking responsibility, guided by professional competence and an internal ethical compass • Leading change and challenging hierarchies to enable holistic care that respects patients’ rights and dignity • Relying on the support of colleagues and management, while facing the risk of exclusion

### 3.5. Ethical Considerations

The study was conducted in accordance with established ethical principles of voluntariness, confidentiality, and informed consent. In line with Swedish legislation, the project did not fall under the scope of the Swedish Ethical Review Act, and therefore, no formal ethical review was required. The design of the project ensured that no sensitive personal data or information regarding legal violations would be collected. Participation was entirely voluntary, and all participants were informed that they could withdraw at any time without providing a reason.

To safeguard confidentiality, all data were anonymized before analysis and publication. Identifying information was removed to protect participants’ identities. This ensured that individuals could contribute to the study without risk of being identified in the reporting of results. In the meta‐synthesis, additional precautions were taken. The AI‐generated synthesis was based solely on the results of the primary analyses, not on original transcripts or direct quotations. This approach ensured that no identifiable personal information was processed during the AI‐assisted phase of the analysis.

The interviews and primary analyses were conducted by master’s‐level students with both clinical experience as nurses and formal training in qualitative research methods. Throughout the project, they received supervision to ensure methodological rigor and reflexive awareness. Their involvement was considered ethically appropriate, as they were equipped to handle the data sensitively and responsibly.

## 4. Results

We identified three themes in nurses’ experiences with rebel nurse leadership in psychiatric care:

• Taking responsibility, guided by professional competence and an internal ethical compass
• Leading change and challenging hierarchies to enable holistic care that respects patients’ rights and dignity
• Relying on the support of colleagues and management, while facing the risk of exclusion

These themes collectively highlight the complex and often contentious nature of rebel nurse leadership in psychiatric care. Such leadership can drive significant improvements in patient care and workplace culture, but it also requires a supportive environment, strong personal conviction, and resilience from the nurses who choose to lead in this way. While the challenges and supports may vary slightly depending on the specific psychiatric care setting, the overarching themes remain consistent. Rebel nurse leaders often face isolation and resistance, require considerable support, and demonstrate personal integrity and professional commitment, all directed toward improving patient care through innovative leadership.

### 4.1. Taking Responsibility, Guided by Professional Competence and an Internal Ethical Compass

Nurses engaging in rebel leadership typically demonstrate a strong professional commitment to improving patient care, a willingness to challenge the status quo, and a personal drive for change. They lead by example through small, everyday actions, showing colleagues new and alternative ways of working in practical terms. Evidence‐based arguments are often used to justify these changes, helping to legitimize new approaches when deviating from established routines.

“*Um, you see that there’s a guideline or a way of working that isn’t functioning very well. Then you start thinking, how can we work instead? And then actually take responsibility for making that change happen…*” Participant 6, general psychiatric inpatient care.

Rebel leaders were described as having a deep understanding of patient needs and demonstrating courage in their convictions, despite potential opposition or challenges. A strong sense of personal and professional responsibility permeated participants’ accounts. These nurses often go beyond the call of duty to advocate for their patients’ best interests, challenging outdated practices and protocols.

“*…it doesn’t always have to mean thinking outside the box, pushing boundaries, or ‘rebelling,’ so to speak. It can also be about daring a little more or trying new things, exploring one’s role as a nurse and one’s role in relation to patients. But I also hope that it encourages others to stand up as well…*” Participant 7, general psychiatric outpatient care.

Taking responsibility for the patient was exemplified by “doing the little extra” and acting swiftly in urgent situations, even beyond normal duties or outside working hours.

“*And I have, among other things, stopped train traffic outside of my working hours because I received a message from a patient standing on the tracks. I couldn’t just say, ‘No, no, but I’m off duty, so… that’s not my responsibility.’*” Participant 1, general psychiatric outpatient care.

Across all care contexts included in the study, rebel leaders shared common traits of resilience and ethical commitment to both nursing practice and the individuals receiving care. This commitment was expressed through a focus on relationship‐building and spending time getting to know the patient properly, even when this went against time‐efficient care models. Their resilience and ethical dedication served as driving forces for initiating change to improve nursing care.

The role of a rebel leader was described as requiring considerable emotional resilience and moral strength. These leaders often faced potential backlash and had to maintain a clear focus on patient welfare, grounded in professional knowledge. This could entail personal sacrifices and require a strong ethical compass to guide their decisions.

“*I was in training or had just qualified as a specialist nurse, and she became a role model or an inspiration for me. She stands up for what is right!*” Participant 4, forensic care.

“*I mean, when it comes to that – daring to stand up for the patients in such matters. And for me, it’s the same. Like, okay, but I’m just doing my job. It doesn’t feel like I’m doing anything beyond that […] Before I even read about what good rebel leadership is, it had always been obvious to me that the patients’ needs are what matter most.*” Participant 5, child and adolescent inpatient care.

### 4.2. Leading Change and Challenging Hierarchies to Enable Holistic Care That Respects Patients’ Rights and Dignity

Rebel leaders play a crucial role in challenging and changing the culture within psychiatric care settings. They advocate for practices that prioritize individual patient needs over rigid adherence to outdated protocols, contributing to broader organizational and cultural shifts. Furthermore, they defend the nursing profession against hierarchical structures and outdated traditions by challenging decisions made by, for example, physicians or management when these decisions do not serve the patient’s best interests. Rebel leaders are willing to face conflict to protect care quality and adapt care to the individual by bending routines and making small adjustments to meet patients’ actual needs, even if this means breaking standard procedures. By identifying problems in daily practice and introducing new initiatives despite resistance, they highlight care deficiencies and propose new working methods to enable improvements.

“*Someone has decided on things that don’t work very well in reality. Rebel leadership arises when there’s an expectation, a silent agreement, that I should always be extremely flexible – I don’t like that.*” Participant 6, forensic care.

Examples of what rebel leadership entails in different contexts include both team dynamics in relation to hierarchies and nursing care in relation to the individual. In general psychiatric inpatient care, this was exemplified by shifting team dynamics to become more collaborative and less hierarchical. In general psychiatric outpatient care, the focus was on enabling more holistic approaches, for example, by adjusting scheduling practices or integrating mental health services with community resources. In child and adolescent psychiatric inpatient care, efforts were made to create a more inclusive environment for families and a less intimidating setting for young patients.

“*Yeah, I remember my first week – there was this young guy who had been admitted, and his mom was coming to visit. He was very anxious and very psychotic. But when she arrived early in the morning, the person responsible for him said, ‘No, he has to come after breakfast,’ to his mom. But I asked, ‘Can’t we just bring in his breakfast so he can eat with his mom? That would probably help him feel calmer.’ And it was like, ‘Oh…we haven’t even thought about that.’ That really stuck with me as a positive moment. These small, small things can be incredibly important.*” Participant 5, general psychiatric inpatient care.

Prioritizing the patient’s perspective over organizational routines by questioning why things are done a certain way, and whether it truly benefits the patient, was a common trait of rebel leadership. In forensic care, such leadership may aim to influence policy and promote nursing practices that respect patient dignity and support rehabilitation over punishment.

“*…one must never forget that we work with people. We can never follow the rules 100% of the time. These unwritten rules exist to be broken sometimes if it makes things easier for the patient – and for the nurse as well, then, at some point, you just have to break them.*” Participant 2, forensic care.

Rebel leaders often strive to foster a more open, learning‐oriented work environment that supports innovation and person‐centered care. Their efforts can lead to improved quality of care and better patient outcomes, such as reduced hospital stays and a decreased need for coercive measures.

“*…one might see deficiencies in the workplace and want to change them. Perhaps one feels unable to stand behind the current state of care and wants to make a change, which in turn leads to a desire to do something new. One wants to improve and develop routines and guidelines within the unit where one works. So, I think it’s very much about one’s own…eh, one’s own vision of the kind of care one wants to provide as a nurse.*” Participant 4, general psychiatric inpatient care.

In general psychiatric inpatient care, rebel leadership often led to improved patient engagement and reduced use of coercive measures, demonstrating the impact of person‐centered approaches. In outpatient care, innovations led by rebel nurses helped streamline processes, reduce waiting times, and improve patient follow‐up, enhancing both patient satisfaction and outcomes. In child and adolescent psychiatric inpatient care, changes focused on making the environment more child‐friendly and improving the effectiveness of therapy by incorporating play and education.

“*…it’s quite sterile, strict, and rather rigid on a ward. And I also reflect on how one can get stuck in the illness, so to speak. I had many discussions with others about this – maybe we could have a little rabbit hutch out in the backyard, so that one could go outside and be in a completely different environment.*” Participant 7 child and adolescent psychiatric inpatient care.

In forensic care, rebel initiatives focused on rehabilitation and reintegration strategies, which were described as improving patient morale and reducing recidivism.

### 4.3. Relying on the Support of Colleagues and Management, While Facing the Risk of Exclusion

Effective rebel leadership is heavily dependent on support from both management and colleagues. Nurses who receive encouragement and backing are more likely to succeed in implementing new practices. Building formal or informal support systems with like‐minded colleagues was described as essential for mentorship and resilience. In such environments, person‐centered care was valued and encouraged, enabling rebel leadership to persist despite challenges.

“*There should be a nurse group for those who want to be rebel leaders, so they can support each other…You need that support because I know I’ve received support from the colleague I see as a rebel nurse. When facing setbacks, it helps to have someone who is on the same page – someone who can encourage you and say, ‘Come on! Keep going! This will work out!’*” Participant 9, general psychiatric outpatient care.

Nurses working in general psychiatric inpatient care emphasized that support from management and colleagues was critical for sustaining rebel leadership. Reinforcement and encouragement enabled the continuation of new practices. In general psychiatric outpatient care, the decentralized nature of the setting meant that support needed to be more proactive and visible to be effective. Additionally, participants noted the importance of endorsements from senior medical staff to gain legitimacy for their actions.

“*No, but I think what’s important is that it’s hard to practice rebel leadership alone in a workplace. I believe you need at least one other person – it doesn’t matter whether it’s a doctor, a nursing assistant, or someone else – just someone who thinks a little differently and can offer support.*” Participant 5, general psychiatric inpatient care.

Support for rebel leadership in child and adolescent psychiatric inpatient care was described as requiring additional layers, including those from families and educational consultants, to successfully implement and maintain new practices. In forensic care, where legal and security concerns dominate, management support was viewed as crucial. Endorsement from leadership could significantly shield rebel leaders from potential backlash in such tightly regulated environments.

“*There needs to be more openness and support from leadership, absolutely, so that you feel backed from above – I truly believe that. And for those who feel they want to be rebellious in their professional role…to push things forward, even when it goes against norms, routines, structures, or care episodes – whatever it may be…*” Participant 2 general psychiatric outpatient care.

Despite the importance of support, nurses who adopted a rebel approach to implement new practices and meet individual patients’ needs often faced significant challenges. These included resistance from colleagues and management, conflicts arising from questioning established routines, and the emotional toll of being isolated or marginalized. A lack of resources and rigid institutional procedures were also described as major barriers.

In general psychiatric inpatient care, nurses often felt isolated in their attempts to drive change. Resistance to new practices came primarily from colleagues and ingrained routines.

“*Because I was at a workplace where I really tried to change things that I felt weren’t working. But I couldn’t get anyone on board, and that even led to me becoming somewhat of an outsider in the group and no longer feeling comfortable at work. The only way to solve it was to adapt and do things like everyone else, just to avoid constantly feeling like I was fighting against the wind.*” Participant 6, general psychiatric inpatient care.

Similar challenges were evident in general psychiatric outpatient care. However, due to the less centralized nature of the setting, support from colleagues was less direct, and administrative expectations added pressure. In child and adolescent psychiatric inpatient care, challenges included institutional resistance as well as skepticism from parents or guardians. In forensic care, the highly structured and regulated environment meant that changes could be perceived as disruptions to safety and protocol. As a result, nurses not only faced isolation but also direct opposition from security‐focused staff.

“*There was a lot of frustration about feeling like no one was listening. I was relatively new and leading a team of mental health nursing assistants who had been working for a long time, and I was a bit younger. It was very difficult. There was an established way of doing things that I didn’t see as either the most ethical or the best for the patients.*” Participant 2 forensic care.

## 5. Discussion

The results describe nurses’ experiences with rebel nurse leadership in psychiatric care as *Taking responsibility, guided by professional competence and an internal ethical compass; Leading change and challenging hierarchies to enable holistic care that respects patients’ rights and dignity; Relying on the support of colleagues and management, while facing the risk of exclusion*.

Overall, our findings suggest that positive deviance, in the form of rebel nurse leadership, may contribute to much‐needed improvements in psychiatric care. While the study design does not allow us to conclude that rebel nurse leadership definitively leads to positive change, our results contribute to research highlighting the potential of positive deviance to improve care quality [18, 19].

Our findings align with previous descriptions of rebel nurse leadership [11, 15], while adding new insights into how this leadership manifests in psychiatric care settings. We argue that rebel nurse leadership must be understood in relation to the “zombification” of the mental health nursing workforce, as described by Lakeman and Molloy [2] and Gabrielsson et al. [1], where nurses are expected to function passively and compliantly—resembling “zombies” rather than autonomous professionals. From this perspective, rebel nurses may be seen as not yet fully zombified—actively resisting this role and encouraging colleagues to reclaim their professional identity.

The actions described in our findings indicate that rebel nurse leadership is not only grounded in professional competence, as previously suggested by de Kok et al. [11, 15], but also rooted in ethical nursing practice. Many participants viewed their actions not as exceptional but as fulfilling the responsibilities all nurses should uphold, aligning with professional standards clearly articulating an expectation that nurses should assume leadership in clinical nursing practice, management, research, and education, and carry personal responsibility and accountability for ethical nursing practice [7]. The notion that practicing core psychiatric nursing values could be seen as “rebellious” points to a concerning shift: If ethical and person‐centered nursing is considered deviant, then the norm must be at odds with the profession’s core principles.

This aligns with Lakeman and Molloy’s [2] argument that mental health nursing has become “zombified,” an occupation that continues to exist but has lost its vital function. Gabrielsson et al. [1] similarly noted that in Swedish psychiatric care, nurses are often hired not for their expertise in mental health nursing but to fulfill the role of medical assistants. In such a context, leading change based on ethical and professional reasoning becomes an act of rebellion.

Our findings suggest that being a rebel nurse leader in psychiatric care is not just about doing things differently for patients, but also about resisting the assumption that nurses should not lead. Leadership itself becomes a deviation from the norm when nurses are not expected, or even permitted, to lead. This reflects broader organizational expectations in which nurses are relegated to subordinate roles, reinforcing the importance of leadership grounded in nursing values and knowledge.

These insights underscore the need for nurses in psychiatric care to advocate for their professional identity and knowledge base. This is consistent with research emphasizing the central role of nurses in ensuring patient safety and care quality [26, 27].

Rebel behavior often stemmed from ethical conflict, specifically, tensions between nurses’ professional values and the organizational constraints they encountered [11, 15]. Given the moral complexities of psychiatric care, including the inherent tension between care and control [13] and between ideals and reality [1], it is unsurprising that rebel nurse leaders were described as defending patients’ rights and dignity.

This study builds on the concept of positive deviance, commonly understood as intentional rule‐breaking that serves the greater good. In health care, such actions are judged by others, often those enforcing rules, based on perceived rightness or wrongness. However, the decision to act lies with the individual nurse [28]. Our findings emphasize the difficulties nurses in psychiatric care face in sustaining professional standards and autonomy, and the importance of managerial and peer support in enabling such leadership. This supports previous research stressing the need to promote professional autonomy among nurses [29].

Organizations that support and retain nurses are better positioned to deliver high‐quality care [26, 30]. While rebel nurse leadership may benefit both services and individuals, a lack of organizational support can lead to significant costs. One critical, often overlooked factor is resistance from other nurses. Lateral violence remains a common experience in the nursing profession and is a contributing factor to burnout and attrition [31]. Nurse managers, therefore, have a responsibility to recognize and support the value of rebel nurse leadership. Creating a psychologically safe, supportive environment allows nurses to uphold professional standards and contribute to person‐centered psychiatric care.

Actually changing nursing practices through rebel nurse leadership might be difficult [20]. Future research could usefully explore whether, and under what conditions, rebel nurse leadership contributes to actual improvements in care processes or outcomes. Establishing such causal links is important for understanding the mechanisms through which everyday acts of leadership influence organizational change, team dynamics, and patient care. To address this, future studies might employ mixed‐methods approaches or intervention‐based research that follows nurses’ initiatives over time.

### 5.1. Strengths and Limitations

A strength of our study is the relatively large number of participants, recruited nationally and from different subspecialties, which contributes to a rich variety of experiences. However, it is up to the reader to determine the transferability of the findings to other contexts through critical reflection [32].

As most of the interviews were conducted digitally, we acknowledge that this format may have shaped the interaction and participants’ comfort. Being interviewed from a familiar environment may have reduced social pressure and supported openness, while the lack of in‐person presence may have limited access to nonverbal cues and affected the depth of rapport. A limitation of the study is the variation in interview duration; the shorter interviews may have provided less opportunity for in‐depth elaboration, which may have influenced the richness of the data. We also note that interviews conducted within forensic psychiatric care were generally shorter than those in other specialties, which may be explained by the fact that participants in this setting were younger, less experienced, and less often specialist‐trained. However, all interviews contributed relevant material.

Most interviews were conducted by master’s‐level psychiatric nursing students with clinical experience and formal training in qualitative methods. Their professional backgrounds and levels of experience may have influenced how questions were asked and how follow‐up probes were used. To support reflexivity, the interviewers received regular supervision from experienced qualitative researchers and engaged in ongoing discussions about their assumptions and preunderstandings.

The use of generative AI in the analytic process constitutes both a strength and a limitation of this study. It is important to emphasize that the authors take full responsibility for the interpretation of the findings presented in this paper. While the use of generative AI in qualitative analysis enables researchers to critically engage with AI‐generated outputs and derive meaningful insights, while maintaining an appropriate analytical distance [33], it is also important to recognize that AI may fail to identify themes that human analysts would detect [34]. In this study, the AI‐generated meta‐synthesis was treated as one possible interpretation, which was then critically reviewed and revised by the human research team. The generated themes were compared with the findings from the separate analyses and subjected to repeated discussions within the research group. The final themes differed substantially from those initially suggested by GPT‐4 and were developed through an iterative process of human interpretation, critical reflection, and validation against the original analyses. For transparency, the original prompt and AI‐generated output are available upon request from the corresponding author.

## 6. Conclusion

This study contributes to the growing body of research on positive deviance in health care by exploring how rebel nurse leadership operates within psychiatric care. It demonstrates that when nurses act in ways that challenge norms, grounded in professional competence and ethical conviction, they can foster person‐centered, ethically sound, and relationally attuned care, even in environments that tend to suppress nursing autonomy. In doing so, this paper reframes acts of resistance not as defiance, but as expressions of professional responsibility.

Our findings highlight a troubling tension: When standard psychiatric nursing values must be enacted “under the radar,” it signals that those values are no longer part of the norm. Rebel nurse leadership, then, is not an outlier behavior but a necessary response to organizational cultures that marginalize the nursing perspective. By naming and legitimizing this leadership, we contribute a conceptual and empirical foundation for recognizing nurses as key change agents in psychiatric care despite systemic barriers. The study also emphasizes the need for supportive infrastructures that protect and empower nurses who strive to uphold their ethical and professional mandates.

## Author Contributions

Sebastian Gabrielsson and Johanna Salberg: conceptualization, formal analysis, methodology, investigation, supervision, and writing–original draft preparation. Git‐Marie Ejneborn Looi: conceptualization, formal analysis, methodology, investigation, supervision, and writing–review and editing. Annika Ahlblom, Ylva Kortekaas Genstrand, Anneli Björkdahl, Jessica Östling, Susanne Berg, Nina Lakso, Mattias Larsson, and Johanna Salberg: formal analysis, investigation, and writing–review and editing.

## Funding

This work received no specific funding.

## Conflicts of Interest

The authors declare no conflicts of interest.

## Data Availability

Research data are not shared.
